# Routine chest x-rays in intensive care units: a systematic review and meta-analysis

**DOI:** 10.1186/cc11321

**Published:** 2012-04-27

**Authors:** Anusoumya Ganapathy, Neill KJ Adhikari, Jamie Spiegelman, Damon C Scales

**Affiliations:** 1 Department of Critical Care Medicine, Sunnybrook Health Sciences Centre; Interdepartmental Division of Critical Care, University of Toronto; 2075 Bayview Avenue, Toronto ON M4N 3M5, Canada; 2 Department of Critical Care Medicine and Sunnybrook Research Institute, Sunnybrook Health Sciences Centre; Interdepartmental Division of Critical Care, University of Toronto; 2075 Bayview Avenue, Toronto ON M4N 3M5, Canada; 3 Department of Medicine, Division of Critical Care Medicine, Humber River Regional Hospital, 2111 Finch Avenue West, Toronto ON M3N 1N1, Canada

## Abstract

**Introduction:**

Chest x-rays (CXRs) are the most frequent radiological tests performed in the intensive care unit (ICU). However, the utility of performing daily routine CXRs is unclear.

**Methods:**

We searched Medline and Embase (1948 to March 2011) for randomized and quasi-randomized controlled trials (RCTs) and before-after observational studies comparing a strategy of routine CXRs to a more restrictive approach with CXRs performed to investigate clinical changes among critically ill adults or children. In duplicate, we extracted data on the CXR strategy, study quality and clinical outcomes (ICU and hospital mortality; duration of mechanical ventilation and ICU and hospital stay).

**Results:**

Nine studies (39,358 CXRs; 9,611 patients) were included in the meta-analysis. Three trials (N = 870) of moderate to good quality provided information on the safety of a restrictive routine CXR strategy; only one trial systematically assessed for missed findings. Pooled data from trials showed no evidence of effect of a restrictive approach on ICU mortality (risk ratio [RR] 1.04, 95% confidence interval [CI] 0.84 to 1.28, *P *= 0.72; two trials, N = 776), hospital mortality (RR 0.98, 95% CI 0.68 to 1.41, *P *= 0.91; two trials, N = 259), ICU length of stay (weighted mean difference [WMD] -0.86 days, 95% CI -2.38 to 0.66 days, *P *= 0.27; three trials, N = 870), hospital length of stay (WMD -2.50 days, 95% CI -6.62 to 1.61 days, *P *= 0.23; two trials, N = 259), or duration of mechanical ventilation (WMD -0.30 days, 95% CI -1.48 to 0.89 days, *P *= 0.62; three trials, N = 705). Adding data from six observational studies, one of which systematically screened for missed findings, gave similar results.

**Conclusions:**

This meta-analysis did not detect any harm associated with a restrictive chest radiograph strategy. However, confidence intervals were wide and harm was not rigorously assessed. Therefore, the safety of abandoning routine CXRs in patients admitted to the ICU remains uncertain.

## Introduction

Physicians often order routine daily antero-posterior chest x-rays (CXRs) for patients in intensive care units (ICUs) due to concerns about the severity of cardiopulmonary illness and complexity of medical interventions [1] and for detection of complications associated with indwelling devices, such as endotracheal tubes and central venous catheters. The frequency of complications, such as device malpositioning or pneumothoraces, has led some guidelines to recommend routine CXRs for all patients with acute cardiopulmonary problems or receiving mechanical ventilation [2]. Advantages of routine CXRs may include prompt detection and thus earlier treatment of clinically unsuspected abnormalities, documentation of disease progression or response to therapy, and educational value for trainees [3,4]. In contrast, a restrictive strategy limits CXRs to specific clinical indications, such as a change in clinical status or following certain procedures. Arguments for adopting a restrictive approach include variable interpretation of CXRs depending on clinician and patient factors, low incidence of clinically unsuspected abnormalities, potential harm arising from unnecessary treatment of minor or false positive findings, cost, radiation exposure and adverse events arising from repositioning of the patient to obtain the CXR [5,6].

Our objective was to systematically review the available literature evaluating the effect on clinical outcomes of abandoning routine CXRs in favor of a more restrictive approach.

## Materials and methods

We conducted our study following recommendations from the Preferred Reporting Items for Systematic Reviews and Meta-Analysis (PRISMA) statement [7] and the Meta-analysis of Observational Studies in Epidemiology (MOOSE) Group [8].

### Search Strategy

We searched MEDLINE and EMBASE for relevant articles in any language published between 1948 and March 2011 and limited the search to humans. The MEDLINE search strategy was 'radiography, thoracic' AND 'intensive care units or critical care or critical illness'; the EMBASE strategy was 'radiography, thorax' AND 'intensive care unit or intensive care or critical illness' AND 'daily or day or routine'. We reviewed bibliographies of review articles and all included studies to identify additional articles.

### Study Selection

Three authors (AG, JS, DS) independently selected studies for inclusion if they were randomized or quasi-randomized (for example, alternate allocation or by medical record number) controlled trials or before-after observational studies of restrictive versus routine CXR ordering in patients admitted to adult or pediatric medical or surgical ICUs. To be eligible for inclusion, studies had to report our primary outcome of ICU mortality or one of our secondary outcomes (hospital mortality, ICU length of stay, hospital length of stay, duration of mechanical ventilation). In the case of duplicate data publication (several studies with overlapping samples), we included only the most recent study. We defined the 'routine CXR' strategy as daily CXRs and the 'restrictive CXR' strategy to mandate CXRs only when a problem was clinically suspected or following certain procedures.

### Data Extraction

Three authors (AG, JS, DS) independently extracted the following data using a standardized spreadsheet: year of study, location of study, type of ICU (adult medical, surgical, or mixed; pediatric), patient selection criteria and patient outcomes (as listed above) in the restrictive and routine CXR strategies. A fourth author (NA) verified outcomes data. Disagreements between reviewers were resolved by consensus.

### Study Quality

Three authors (AG, DS, NA) assessed the randomized controlled trials (RCTs) and quasi-RCTs for quality based on the following factors: method of allocation, allocation concealment, blinded outcomes assessment and losses to follow-up. We adapted the Newcastle-Ottawa Quality Assessment Scale to evaluate the before-after observational studies [9].

### Data Analysis

Our primary outcome was ICU mortality; secondary outcomes were hospital mortality, ICU and hospital length of stay, and duration of mechanical ventilation. Our main analyses included data from RCTs and quasi-RCTs. We also conducted sensitivity analyses of all outcomes combining results of RCTs, quasi-RCTs, and before-after observational studies.

Meta-analyses were performed using Review Manager 5.1 software (Cochrane Collaboration, Oxford, UK) using random-effects models [10], which generally provide more conservative estimates of treatment effect in the presence of heterogeneity [10]. Risk ratios (RR) were calculated for binary outcomes and weighted mean differences (WMD) for continuous outcomes, along with 95% confidence intervals (CI). All statistical tests were two-sided, with *P *≤ 0.05 interpreted as statistically significant. We measured heterogeneity as *I^2^*, the percentage of total variation across studies owing to between-study heterogeneity rather than chance [11], and used suggested thresholds for low (*I^2 ^*= 25% to 49%), moderate (*I^2 ^*= 50% to 74%) and high (*I^2 ^*≥ 75%) [12] values. Given the small number of studies included in the meta-analysis, exploration of variation in treatment effects using meta-regression was not possible.

One study reported a cluster RCT of 21 ICUs in 18 hospitals that mandated cross-over between the restrictive and routine CXR strategies [13]. We adopted the approach recommended by the Cochrane Collaboration [14] to incorporate the results of this trial by adjusting the variance of the outcome measure for the design effect due to clustering. The design effect was calculated as 1 + ((m - 1) × ICC), where m is the number of observations per cluster and ICC is the intracluster correlation coefficient. The ICC is a measure of the correlated (non-independent) nature of observations made within a cluster due to the similarity of individuals in a cluster [14]. It is calculated as between-cluster variance divided by total variance, where total variance = within-cluster variance + between-cluster variance. ICC may take on any value from 0 to 1. A very small value of ICC implies that individuals within a cluster behave very dissimilarly; in other words, the variance within the clusters is much greater than the variance between the clusters. When ICC = 0, the sample size requires no adjustment because the individuals within each cluster have zero similarity to each other. As ICC approaches 1, the effective sample size is reduced progressively. When ICC = 1, all individuals in a given cluster behave identically, and the effective sample size equals the number of clusters.

**Figure 1:**
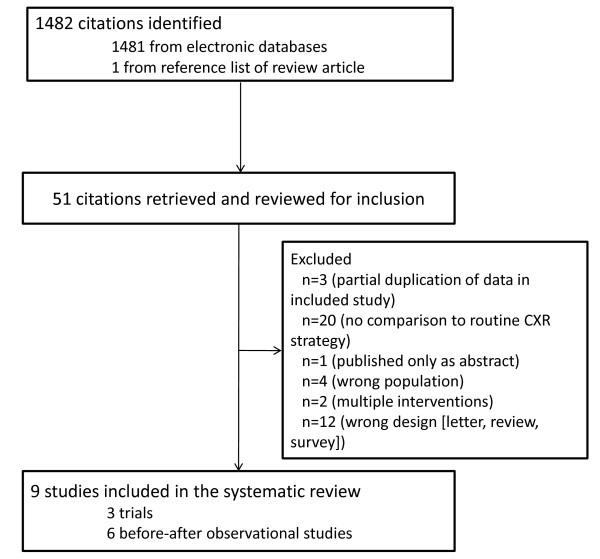
**Flow of studies through the systematic review**.

For the cluster RCT, we estimated a cluster size of 40 from Figure 1 of the publication [13] (that is, approximately 20 patients per cross-over period per ICU) and an ICC of 0.01 (actual ICC not reported), which gives a design effect of 1.39. For binary outcomes, the sample size and number of events were divided by the design effect. For continuous outcomes, the sample size was divided by the design effect, but the mean and standard deviation were unchanged. We used the adjusted sample size for this trial when reporting the number of patients in each meta-analysis. Sensitivity analyses using values of 0.05 or 0 for the ICC did not qualitatively change any results (Additional file 1). We were unable to account for possible treatment-order effects arising from the cross-over aspect of this trial's design because of limitations of the data provided.

## Results

### Characteristics of included studies

Our search identified 1,482 citations, of which we retrieved 51 citations for more detailed evaluation (Figure 1). Two RCTs [13,15], one quasi-randomized trial [16] (Table 1) and six before-after observational studies [17-22] (Table 2) met selection criteria. We excluded 42 studies because of partial duplication of data included in the review [23-25]; lack of comparison to routine CXR strategy [26-45]; publication as abstract only [46]; multiple interventions [47,48]; wrong population [49-52] and wrong design [letter, review, survey] [1,53-63].

**Table 1:** Characteristics and methodological quality of randomized and quasi-randomized controlled trials of restrictive versus routine CXR strategies.

Study	ICU Population	Patient selection criteria	Location	Total patients (n)	CXRs/patients, restrictive group (n/n)	CXRs/patients, routine group (n/n)	Outcomes used in meta-analysis	Method of allocation	Allocation concealment	Blinded outcomes assessment^a^	Zero losses to follow up
Krivopal *et al.*, 2003 [16]	Medical, Adult	Ventilated for 48 to 72 hours Exclusion criteria: reintubated, transferred from other centers	USA	94	226/51	293/43	ICU and hospital mortality ICU and hospital length of stay Duration of mechanical ventilation	Quasi-randomization based on last digit of medical record number	No	Not specified	Yes
Clec'h *et al.*, 2007^b ^[15]	Medical-surgical, Adult	Ventilated for ≥ 48 hours Exclusion criteria: reintubated, tracheostomy, or withdrawal of life support	France	165	94/81	885/84	ICU and hospital mortality ICU and hospital length of stay	Computer-generated random number table	Not reported	Not specified	Yes
Hejblum *et al.*, 2009 [13]	Medical-surgical, Adult	Ventilated for ≥ 2 days	France	611, 849^c^	3,148/306, 425^c^	4,607/305, 424^c^	ICU mortality ICU length of stay Duration of mechanical ventilation	Computer-generated	Not reported ('open-label with respect to allocation concealment')	Not specified	Yes

^a^Radiologists and clinicians were not blinded to the group when interpreting films; ^b^In this trial all patients had daily CXRs, but results in the restrictive group were concealed from clinicians, who were free to order CXRs as needed. ^c^The first sample size is adjusted for the design effect due to clustering [14], assuming intracluster correlation 0.01 (design effect 1.39); the second is the unadjusted sample size as reported in the study. CXR, chest X-ray; n, number.

**Table 2:** Characteristics of included observational (before-after) studies of restrictive versus routine CXR strategies.

Study	ICU Population	Patient selection criteria	Location	Total patients (n)	CXRs/patients, restrictive group (n/n)	CXRs/patients, routine group (n/n)	Outcomes used in meta-analysis
Rao *et al.*, 1997 [17]^a^	Post cardiac surgery	Exclusion criteria: ICU length of stay > 36 hours, death within 36 hours	UK	200	36/100	304/100	Hospital length of stay
Price *et al.*, 1999 [18]^b^	Pediatric	Exclusion criterion: Cardiothoracic surgical patients	USA	3,427	5,939/1,588	10,585/1,839	ICU and hospital length of stay^c^ Duration of mechanical ventilation^c^
Leong *et al.*, 2000 [19]^d^	Post cardiac surgery	All patients included	USA	200	334/100	520/100	Hospital mortality ICU and hospital length of stay Duration of mechanical ventilation
Krinsley *et al.*, 2003 [20]	Medical-surgical, Adult	All patients included	USA	2,564	2,298/1,267	3,093/1,297	Hospital mortality ICU length of stay Duration of mechanical ventilation
Graat *et al.*, 2007 [21]	Medical-surgical, Adult	Exclusion criteria: Readmissions	Netherlands	1,376	1,115/622	3,194/754	ICU and hospital mortality
Hendriske *et al.*, 2007 [22]	Medical-surgical, Adult	Exclusion criteria: Cardiothoracic surgical and neurosurgical patients	Netherlands	736	907/250	1,780/486	Hospital mortality

^a^The study included one group with routine CXRs, a second group with less frequent routine CXRs, and a third group with CXRs ordered only as clinically indicated. We abstracted data from the first and third groups; ^b^This study included a control phase with routine CXRs, an evaluative phase with a small convenience sample of patients with routine CXRs in which the investigators studied the impact of routine CXRs on patient management, and a post intervention phase with no routine CXRs. We abstracted data from the first and third phases; ^c^Outcomes data were abstracted from Figure 5; ^d^This study included a control group with routine daily CXRs, a second group with CXRs ordered when clinically indicated and routine CXRs only on admission and after extubation (two months after the change was implemented), and a third group with the same CXR strategy as the second group studied 4 years after the change was implemented. We abstracted data from the first and third groups. CXR, chest X-ray; n, number.

The nine studies included a total of 39,358 CXRs done on 9,611 patients from the United States, Canada, France, The Netherlands and Germany. One study [18] was conducted in a pediatric ICU; the rest were conducted in adult medical, surgical or combined medical-surgical ICUs. One study [13] was a cluster RCT including 849 patients, with an effective sample size of 611 after adjusting for clustering, assuming an ICC of 0.01.

Overall study quality was moderate to good for trials (Table 1) but poor to moderate for before-after observational studies (Table 3). Observational studies were often at risk for selection bias (that is, enrollment of non-consecutive patients) and secular trends in outcomes unrelated to the intervention. Both trials and observational studies generally did not blind radiologists to the CXR strategy in place at the time the individual CXR was obtained; clinicians and assessors of other outcomes were similarly unblinded. Only two studies (one RCT [15] and one before-after observational study [22]) mandated routine CXRs (with results concealed from clinicians) to screen for missed findings in patients in the restrictive CXR group.

**Table 3:** Newcastle-Ottawa quality assessment scale for before-after observational studies.

STUDY	SELECTION^a^				COMPARABILITY^b^	OUTCOME^c^		
	**Representativeness of the exposed cohort**	**Selection of the non-exposed cohort**	**Ascertainment of exposure**	**Outcome of interest not present at the start of study**	**Cohorts comparable on the basis of design or analysis**	**Assessment of outcome**	**Adequacy of duration of follow-up**	**Adequacy of completeness of follow-up**
Rao *et al.*, 1997 [17]		*	*	*	*			
Price *et al.*, 1999 [18]	*	*	*	*	*		*	
Leong *et al.*, 2000 [19]		*	*	*	*		*	
Krinsley *et al.*, 2003 [20]	*	*	*	*	*		*	
Graat *et al.*, 2007 [21]	*	*	*	*	*			
Hendrikse *et al.*, 2007 [22]	*	*	*	*	*	*		

Refer to reference [9] for a description of Newcastle-Ottawa Quality Assessment Scale for cohort studies. In general, more stars denote higher quality. A study can be awarded a maximum of one star for each item within the 'Selection' and 'Outcome' categories. **^a^**A maximum of four stars can be given for 'Selection'. 'Representativeness' is awarded a star if the cohort is truly or somewhat representative of the population of interest. For selection of the non-exposed cohort, a star is awarded if it is drawn from the same population as the exposed cohort. Exposure is satisfactorily ascertained if data are collected from a secure record. ^b^A maximum of two stars can be given for 'Comparability', one each for controlling of two important confounders in either the design or analysis phase. **^c^**A maximum of three stars can be given for 'Outcome'. 'Assessment of outcome' is awarded a star if the outcomes were assessed by independent blind assessment or record linkage. The duration of follow-up was considered adequate if it was long enough for the outcomes to occur. Completeness of follow-up was considered adequate if all patients were accounted for or if the number lost to follow-up was sufficiently low to be unlikely to introduce bias.

### Mortality outcomes

**Figure 2:**
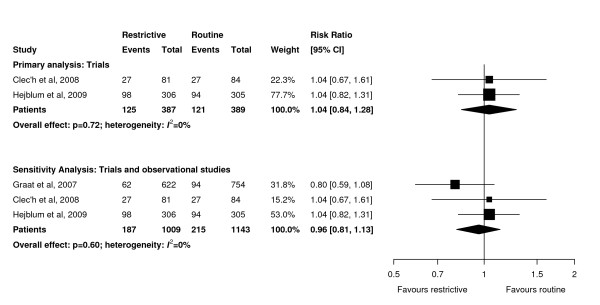
**Effect of a restrictive versus routine chest x-ray strategy on intensive care unit mortality among trials (primary analysis) and trials and observational studies (sensitivity analysis)**. The number of events and sample size of Hejblum *et al. *[13] have been adjusted for clustering (see Methods for details). Weight is the relative contribution of each study to the overall estimate of treatment effect on a log scale using a random effects model.

**Figure 3:**
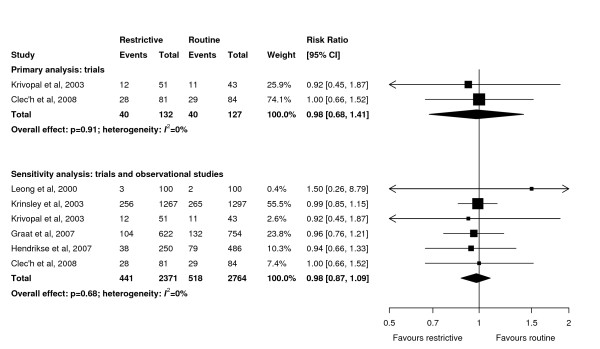
**Effect of a restrictive versus routine chest x-ray strategy on hospital mortality among trials (primary analysis) and trials and observational studies (sensitivity analysis)**. Weight is the relative contribution of each study to the overall estimate of treatment effect on a log scale using a random effects model.

Pooled data showed that the primary outcome of ICU mortality (RR 1.04, 95% CI 0.84 to 1.27, *P *= 0.78; two trials, N = 776; Figure 2) was similar in the restrictive and routine CXR groups. Results were similar for hospital mortality (RR 0.98, 95% CI 0.68 to 1.41, *P *= 0.91; two trials, N = 259; Figure 3).

### Other secondary outcomes

**Figure 4:**
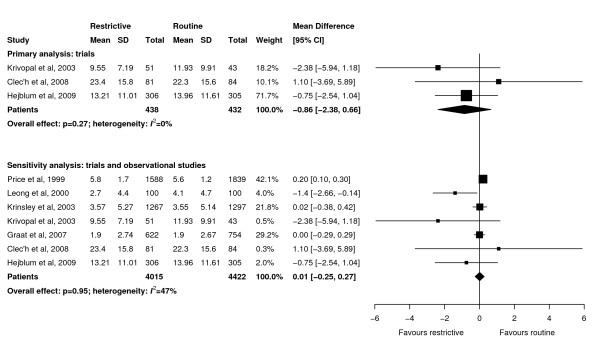
**Effect of a restrictive versus routine chest x-ray strategy on intensive care unit length of stay in days among trials (primary analysis) and trials and observational studies (sensitivity analysis)**. The sample size of Hejblum *et al. *[13] has been adjusted for clustering (see Methods for details). Weight is the relative contribution of each study to the overall estimate of treatment effect using a random effects model.

**Figure 5:**
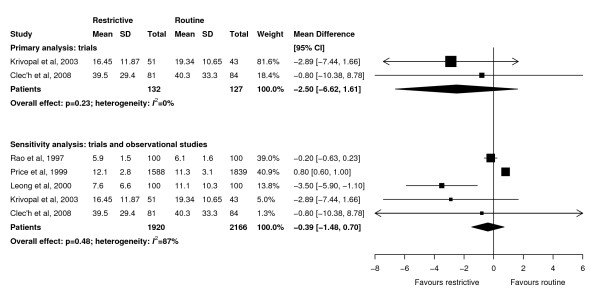
**Effect of a restrictive versus routine chest x-ray strategy on hospital length of stay in days among trials (primary analysis) and trials and observational studies (sensitivity analysis)**. Weight is the relative contribution of each study to the overall estimate of treatment effect using a random effects model.

**Figure 6:**
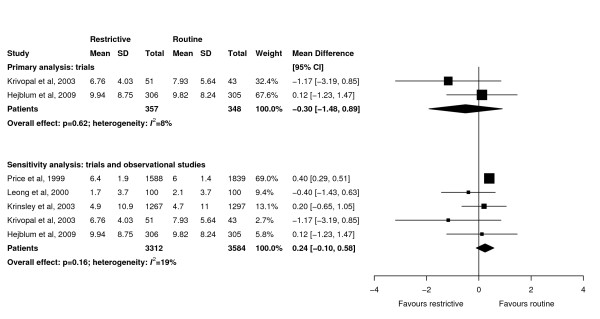
**Effect of a restrictive versus routine chest x-ray strategy on duration of mechanical ventilation in days among trials (primary analysis) and trials and observational studies (sensitivity analysis)**. The sample size of Hejblum *et al. *[13] has been adjusted for clustering (see Methods for details). Weight is the relative contribution of each study to the overall estimate of treatment effect using a random effects model.

Meta-analyses showed that ICU length of stay (WMD -0.86 days, 95% CI -2.38 to 0.66 days, *P *= 0.27; three trials, N = 870; Figure 4), hospital length of stay (WMD -2.50 days, 95% CI -6.62 to 1.61 days, *P *= 0.23; two trials, N = 259; Figure 5), and duration of mechanical ventilation (WMD -0.30 days, 95% CI -1.48 to 0.89 days, *P *= 0.62; three trials, N = 870; Figure 6) were not significantly different between groups. There was little heterogeneity in all analyses restricted to trials (*I^2 ^*≤ 8%).

### Sensitivity analyses

Sensitivity analyses combining results of trials and observational studies showed no difference between groups for ICU mortality (RR 0.96, 95% CI 0.81 to 1.13, *P *= 0.60; three studies, N = 2,152; Figure 2), hospital mortality (RR 0.98, 95% CI 0.87 to 1.09, *P *= 0.68; six studies, N = 5,135; Figure 3), ICU length of stay (WMD 0.01 days, 95% CI -0.25 to 0.27 days, *P *= 0.95; seven studies, N = 8,437; Figure 4), hospital length of stay (WMD -0.39 days, 95% CI -1.48 to 0.70, *P *= 0.48; five studies, N = 4,086; Figure 5) and duration of mechanical ventilation (WMD 0.24 days, 95% CI -0.10 to 0.58 days, *P *= 0.16; five studies, N = 6,896; Figure 6). In the sensitivity analyses, there was little heterogeneity for ICU or hospital mortality (*I^2 ^*= 0% for both) or duration of mechanical ventilation (*I^2 ^*= 19%), but there was moderate heterogeneity for ICU length of stay (*I^2 ^*= 47%) and substantial heterogeneity for hospital length of stay (*I^2 ^*= 87%). Additional sensitivity analyses using values of 0.05 and 0 for the ICC of the cluster RCT [13] did not qualitatively change any results (see Additional file 1).

## Discussion

We identified only three trials that evaluated the effects on clinical outcomes in critically ill adults and children of a restrictive strategy of obtaining CXRs only when abnormalities are clinically suspected compared to routine daily CXRs. We found no differences in mortality when results of trials were combined, or when results of an additional six before-after observational studies were also considered. Similarly, duration of mechanical ventilation and ICU and hospital lengths of stay were similar for both CXR strategies.

Strengths of our systematic review include a comprehensive search strategy and broad inclusion criteria, with consideration of both RCTs and quasi-RCTs and before-after observational studies. Although we searched electronic biobliographic databases with no restriction on publication date, studies included in the systematic review were all published in the last 15 years and, therefore, are likely representative of current ICU practices. Our study is limited by small sample size, moderate methodological quality and heterogeneity of included studies. Only three trials evaluated the safety of a restrictive versus routine CXR strategy; the combined sample size of these trials would only have sufficient power to detect an implausibly large difference in our primary outcome. Despite the absence of statistical heterogeneity in our analyses restricted to trials, all of which excluded patients mechanically ventilated for < 48 hours, there is likely substantial remaining clinical heterogeneity related to severity of illness and surgical status, for example.

Several studies have evaluated a restrictive CXR strategy with hypotheses that costs and radiation exposure would be reduced. The restrictive strategy can substantially reduce the number of CXRs and thus may reduce costs [18,22] but the anticipated cost-savings have varied greatly [29,31,42]. With respect to radiation exposure, levels measured at ICU nursing stations have been shown to be below the maximum permissible for non-occupational workers [64,65]. Although exposure to patients from a single CXR is felt to pose minimum risk, the cumulative exposure is thought to be significant due to the high frequency of this test [66]. In contrast, the main short-term risk of a restrictive CXR strategy in critically ill patients is the potential for a missed or delayed diagnosis, leading to delayed treatment and thus additional morbidity and mortality. We were unable to estimate the number of missed diagnoses or delays in management of complications in the restrictive CXR group because only two studies [15,22] had a surveillance strategy for these patients.

The major design challenge for studies of different CXR ordering strategies is to overcome ascertainment bias, which may lead to more complications (including clinically non-apparent complications) being detected in the routine CXR group if surveillance CXRs are not obtained in the restrictive group. Complications may thus be underestimated in the restrictive CXR group. These study challenges can be overcome by obtaining surveillance CXRs (results blinded to clinicians) in the restrictive group to determine the delay between radiographically apparent and clinically detected complications. Other design challenges include ensuring blinding of those adjudicating radiological and clinical outcomes subject to ascertainment bias (for example, pneumonia) and standardization of definitions of complications. Furthermore, establishing non-inferiority for clinical endpoints in a RCT comparing CXR strategies requires a large sample size and modifications to the usual approach to hypothesis-testing [67]. An alternative approach would be to design a non-inferiority trial powered on the surrogate outcome of delayed or missed diagnoses. The heterogeneity of patients studied to date underscores the importance of a non-inferiority trial that tests the hypothesis that the restrictive CXR strategy does not increase harm and that is adequately powered to examine pre-specified subgroup effects. Finally, even if trials demonstrate the non-inferiority of the restrictive CXR strategy, ICUs implementing this strategy should have immediate access to clinicians who can reliably identify patients requiring urgent CXRs, interpret images that are promptly obtained and processed, and act rapidly on any findings requiring intervention [68].

A recent study using a web-based Delphi technique explored common indications for CXRs in critically ill patients, as determined by 82 French intensivists [69]. Consensus was reached that CXRs should be considered routinely after certain procedures (for example, insertion of endotracheal tube, subclavian or internal jugular central venous catheter, pulmonary artery catheter, temporary transvenous pacing lead, chest tube) and during invasive mechanical ventilation for acute respiratory distress syndrome or status asthmaticus. There was also consensus that CXRs were unnecessary in patients with an existing nasogastric tube for enteral feeding or with an existing central venous catheter previously documented to be correctly positioned in the superior vena cava. However, there was no consensus on the need for routine CXRs in intubated patients. The clinical impact and safety of implementing these consensus recommendations have not been evaluated, but the findings of our review suggest no glaring safety concerns with a restrictive CXR strategy in critically ill patients while underscoring the need for additional large randomized trials to establish definitively the non-inferiority of this approach.

## Conclusions

Pooling the results of three trials alone or combined with six before-after observational studies that evaluated the safety of abandoning routine CXRs in favor of a more restrictive approach did not detect harm. However, given the small overall sample size and infrequent systematic evaluation of missed findings in the restrictive strategy group, the safety of abandoning routine CXRs in critically ill patients remains uncertain and mandates further investigation.

## Key messages

• Few trials have compared restrictive versus routine CXRs in the ICU.

• Combined results from three trials did not show harm from a restrictive CXR strategy in the ICU; results were similar when six before-after observational studies were also considered.

• However, given the small overall sample size and limited data on the effects of missed findings on routine CXRs, the safety of abandoning routine CXRs in critically ill patients remains uncertain.

## Abbreviations

CI: confidence interval; CXR: chest x-ray; ICC: intracluster correlation coefficient; ICU: intensive care unit; RCT: randomized controlled trial; RR: risk ratio; WMD: weighted mean difference.

## Competing interests

The authors declare that they have no competing interests.

## Authors' contributions

AG screened studies for inclusion, abstracted data, analyzed data, and drafted the manuscript. JS screened studies for inclusion, abstracted data, analyzed data, and drafted the manuscript. NA verified outcomes data, assessed study quality, analyzed data, and helped to draft the manuscript. DS screened studies for inclusion, abstracted data, analyzed data, and helped to draft the manuscript. AG, NA, and DS critically revised the manuscript for important intellectual content. All authors read and approved the final manuscript.

## Supplementary Material

Additional file 1 **Sensitivity analyses for the effect of a restrictive versus routine chest x-ray strategy on patient outcomes, assuming different values of ICC for the cluster randomized trial**. The file contains two tables with meta-analytic data from sensitivity analyses that assume an intracluster correlation coefficient of 0 or 0.05 for the cluster RCT [13], instead of 0.01 as assumed in the main analyses. Click here for file

## Acknolwedgments

We thank Dr Ruxandra Pinto for assistance with the statistical analysis and with the figures. This study received no funding. DS is supported by a New Investigator Award from the Canadian Institutes of Health Research, which had no role in the design or conduct of the study or decision to submit for publication. Preliminary results have been presented in abstract form (Spiegelman J, Pinto R, Adhikari N, Scales D. Should routine daily chest radiography in Intensive Care Units (ICUs) be abandoned? A systematic review [abstract]. *Am J Respir Crit Care Med *2009, **179: **A1618).
