# Supplementary material for: Routine chest x-rays in intensive care units: a systematic review and meta-analysis
Source: Crit Care. 2012 Apr 27;16(2):R68. doi: 10.1186/cc11321 (PMC3681397; doi:10.1186/cc11321)
Supplement: Additional file 1 — Sensitivity analyses for the effect of a restrictive versus routine chest x-ray strategy on patient outcomes, assuming different values of ICC for the cluster randomized trial. The file contains two tables with meta-analytic data from sensitivity analyses that assume an intracluster correlation coefficient of 0 or 0.05 for the cluster RCT [13], instead of 0.01 as assumed in the main analyses. [file cc11321-S1.PDF]

Sensitivity analyses for the effect of a restrictive vs. routine chest x-ray strategy on patient outcomes, assuming different values of ICC for the cluster randomized trial*

**Outcomes with ICC = 0**	**Results**
Trials only	
ICU mortality	RR 1.04, 95% CI 0.86 to 1.24, p=0.70; I^2 = 0%; 2 trials, N=1014
ICU length of stay	WMD -0.84 days, 95% CI -2.18 to 0.51 days, p=0.22; I^2 = 0%; 3 trials, N=1108
Duration of mechanical ventilation	WMD -0.25 days, 95% CI -1.39 to 0.89 days, p=0.67; I^2 = 16%; 2 trials, N=943
Trials and observational studies	
ICU mortality	RR 0.97, 95% CI 0.82 to 1.14, p=0.67; I^2 = 5%; 3 studies, N=2390
ICU length of stay	WMD 0.00 days, 95% CI -0.27 to 0.27 days, p=0.99; I^2 = 48%; 7 studies, N=8675
Duration of mechanical ventilation	WMD 0.24 days, 95% CI -0.09 to 0.57 days; p=0.16; I^2 = 20%; 5 studies, N=7134

**Outcomes with ICC = 0.05**	**Results**
Trials only	
ICU mortality	RR 1.04, 95% CI 0.80 to 1.37, p=0.76; I^2 = 0%; 2 trials, N=453
ICU length of stay	WMD -0.93 days, 95% CI -2.86 to 1.00 days, p=0.35; I^2 = 0%; 3 trials, N=547
Duration of mechanical ventilation	WMD -0.51 days, 95% CI -1.91 to 0.90 days, p=0.48; I^2 = 0%; 2 trials, N=382
Trials and observational studies	
ICU mortality	RR 0.93, 95% CI 0.76 to 1.13, p=0.46; I^2 = 0%; 3 studies, N=1829
ICU length of stay	WMD 0.02 days, 95% CI -0.23 to 0.28 days, p=0.86; I^2 = 44%; 7 studies, N=8114
Duration of mechanical ventilation	WMD 0.24 days, 95% CI -0.10 to 0.59 days, p=0.17; I^2 = 18%; 5 studies, N=6573

* Hejblum G, Chalumeau-Lemoine L, Ioos V, Boelle PY, Salomon L, Simon T, Vibert JF, Guidet B: **Comparison of routine and on-demand prescription of chest radiographs in mechanically ventilated adults: a multicentre, cluster-randomised, two-period crossover study.** Lancet 2009, **374**(9702):1687-1693.

This trial does not contribute data to meta-analyses of hospital mortality or hospital length of stay, which are therefore not included in the table.

Abbreviations: CI, confidence interval; ICC, intraclass correlation; ICU, intensive care unit; RR, risk ratio; WMD, weighted mean difference
